# Cooperative Metabolism in a Three-Partner Insect-Bacterial Symbiosis Revealed by Metabolic Modeling

**DOI:** 10.1128/JB.00872-16

**Published:** 2017-07-11

**Authors:** Nana Y. D. Ankrah, Junbo Luan, Angela E. Douglas

**Affiliations:** a Department of Entomology, Cornell University, Ithaca, New York, USA; b Department of Molecular Biology and Genetics, Cornell University, Ithaca, New York, USA; Geisel School of Medicine at Dartmouth

**Keywords:** flux balance analysis, genome reduction, metabolic model, “Candidatus Hamiltonella defensa”, nutrient exchange, “Candidatus Portiera aleyrodidarum”, metabolic modeling

## Abstract

An important factor determining the impact of microbial symbionts on their animal hosts is the balance between the cost of nutrients consumed by the symbionts and the benefit of nutrients released back to the host, but the quantitative significance of nutrient exchange in symbioses involving multiple microbial partners has rarely been addressed. In this study on the association between two intracellular bacterial symbionts, “Candidatus Portiera aleyrodidarum” and “Candidatus Hamiltonella defensa,” and their animal host, the whitefly Bemisia tabaci, we apply metabolic modeling to investigate host-symbiont nutrient exchange. Our *in silico* analysis revealed that >60% of the essential amino acids and related metabolites synthesized by “Candidatus Portiera aleyrodidarum” are utilized by the host, including a substantial contribution of nitrogen recycled from host nitrogenous waste, and that these interactions are required for host growth. In contrast, “Candidatus Hamiltonella defensa” retains most or all of the essential amino acids and B vitamins that it is capable of synthesizing. Furthermore, “Candidatus Hamiltonella defensa” suppresses host growth *in silico* by competition with “Candidatus Portiera aleyrodidarum” for multiple host nutrients, by suppressing “Candidatus Portiera aleyrodidarum” growth and metabolic function, and also by consumption of host nutrients that would otherwise be allocated to host growth. The interpretation from these modeling outputs that “Candidatus Hamiltonella defensa” is a nutritional parasite could not be inferred reliably from gene content alone but requires consideration of constraints imposed by the structure of the metabolic network. Furthermore, these quantitative models offer precise predictions for future experimental study and the opportunity to compare the functional organization of metabolic networks in different symbioses.

**IMPORTANCE** The metabolic functions of unculturable intracellular bacteria with much reduced genomes are traditionally inferred from gene content without consideration of how the structure of the metabolic network may influence flux through metabolic reactions. The three-compartment model of metabolic flux between two bacterial symbionts and their insect host constructed in this study revealed that one symbiont is structured to overproduce essential amino acids for the benefit of the host, but the essential amino acid production in the second symbiont is quantitatively constrained by the structure of its network, rendering it “selfish” with respect to these nutrients. This study demonstrates the importance of quantitative flux data for elucidation of the metabolic function of symbionts. The *in silico* methodology can be applied to other symbioses with intracellular bacteria.

## INTRODUCTION

The trait of nutrient overproduction has evolved in diverse bacteria that have entered into intimate associations with animals (1, 2). This trait is cooperative in that the bacterium-derived nutrients enable the animal host to utilize nutrient-poor or unbalanced diets. Symbioses with nutrient-overproducing bacteria have been studied particularly in plant sap-feeding insects, including aphids, whiteflies, leafhoppers, and cicadas. The symbiotic bacteria provide the insect host with substantial amounts of essential amino acids (EAAs), supplementing the EAA-deficient diet of plant sap, and they have also been implicated in B vitamin provisioning (2). These relationships have the following common features: the bacteria are housed in specialized insect cells, generically known as bacteriocytes, and the bacteria are invariably transmitted vertically via the ovary, resulting in shared bacterial-host evolutionary histories, often spanning >100 million years (1–4). The bacterial symbionts tend to have much reduced genomes, resulting from relaxed selection on genes not required in the host and genomic decay linked to their very small effective population sizes (5–7). As a consequence of their small gene contents, the bacteria have very limited functional traits, including the absence of many metabolic pathways found in free-living bacteria (5, 8).

In many plant sap-feeding insects, bacterial EAA provisioning is mediated by a single bacterial taxon, but in other insects, two (or more) bacteria produce complementary sets of EAAs (8). For instance, in most aphids, EAAs are produced by a single symbiont, Buchnera (9), while in spittlebugs, cicadas, and sharpshooters, EAA production is partitioned between two symbiotic bacteria. From an evolutionary perspective, these patterns are related to the multiple routes by which the symbioses compensate for genomic decay of the EAA-biosynthetic capability of the ancestral (primary) symbiont. In various associations (whether with one or more symbionts), the host can contribute to EAA biosynthesis by selective expression in the bacteriocyte of genes that are functionally equivalent to the decaying genes in the primary symbiont, and these compensatory host genes may be of intrinsic (insect) origin or horizontally acquired from bacteria (10–12). In some associations with two bacterial partners, these host-derived contributions are supplemented by the second symbiont, which has evolved to display specific metabolic functions lost by the primary symbiont (3, 5, 8).

The metabolic contribution of the second symbiont in multipartner symbioses is unambiguous where its metabolic gene content perfectly complements that of the primary symbiont (3, 13, 14). In some associations, however, reactions that lead to the synthesis of certain essential amino acids are duplicated between the partners (8). For these associations, a key challenge is to determine the quantitative contributions of the different bacterial partners to host nutrition, especially in relation to EAA biosynthesis.

The difficulties in assigning functions to individual bacterial symbionts in a multipartner association are particularly acute for whiteflies, such as Bemisia tabaci. These plant sap-feeding insects harbor a primary (obligate) symbiont, “Candidatus Portiera aleyrodidarum” (15). Generally the bacteriocytes bearing “Candidatus Portiera aleyrodidarum” also contain one or more other bacteria, known as secondary symbionts (16, 17), but the identities of secondary symbionts vary between related insect taxa, precluding an extended evolutionary time for metabolic coevolution between both primary and secondary symbionts. Genomic analyses suggest that “Candidatus Portiera aleyrodidarum” provides EAAs and carotenoids to the whitefly (18–20). The role of whitefly secondary symbionts is somewhat obscure, with evidence for a protective role against natural enemies and thermal stress (21–23). It has been suggested, however, that the secondary symbionts may provide EAAs and B vitamins for the host (24, 25). However, these proposed metabolic roles are inferred from analysis of genomic data without quantitative consideration of the abundances of the different bacteria or the patterns of metabolic flux in the different partners.

The goal of this study was to establish the quantitative contributions of bacterial symbionts to EAA provisioning in whiteflies by a combination of metabolic modeling (26) and transcriptional and metabolite profiling. We used Bemisia tabaci MEAM1, in which the bacteriocytes bear both the primary symbiont “Candidatus Portiera aleyrodidarum” and the secondary symbiont “Candidatus Hamiltonella defensa.”

## RESULTS

### Overview of metabolic models.

**FIG 1:**
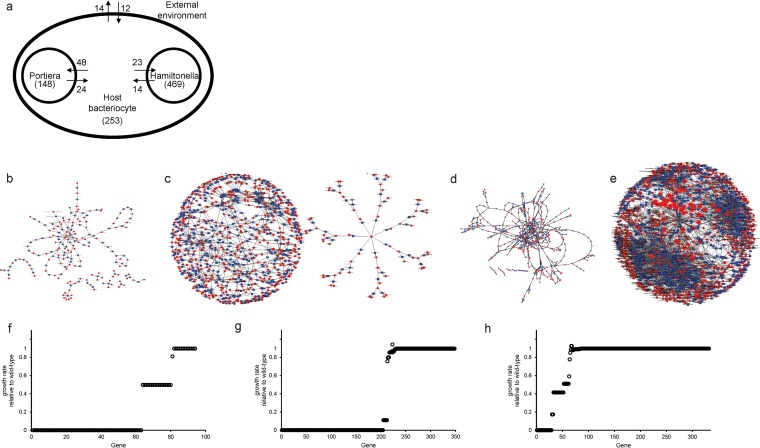
Metabolic models of the three-partner symbiosis between the Bemisia whitefly host and two bacterial symbionts, “Candidatus Portiera aleyrodidarum” and “Candidatus Hamiltonella defensa.” (a) Model structure showing species compartments and metabolites exchanged between compartments. The total number of metabolites in each compartment is shown in parentheses, and the numbers of input and output metabolites for each compartment are displayed alongside the arrows. (b to e) Metabolic-network maps of “Candidatus Portiera aleyrodidarum” *i*NA94 (b), “Candidatus Hamiltonella defensa” *i*NA348 (c), Bemisia *i*NA332 (d), and the integrated three-compartment model *i*NA774 (e) visualized with Cytoscape_v3.4.0. The red circles represent metabolites, and the blue squares represent reactions. (f to h) Genetic robustness of the metabolic networks “Candidatus Portiera aleyrodidarum” *i*NA94 (f), “Candidatus Hamiltonella defensa” *i*NA348 (g), and Bemisia *i*NA332 (h).

The multicompartment bacterial-host model used in this study was constructed to represent the metabolic interactions between bacterial partners and the host as they occur in the host bacteriocyte *in vivo* (Fig. 1a). The genomes of the bacterial partners are highly reduced (“Candidatus Portiera aleyrodidarum,” 0.35 Mb, and “Candidatus Hamiltonella defensa,” 1.7 Mb) (see Fig. S1 in the supplemental material) and, as such, have fewer metabolic genes and reactions than would be expected in a free-living bacterium, such as Escherichia coli (27). Specifically, the “Candidatus Portiera aleyrodidarum” genome includes just 94 metabolism genes, supporting a metabolic network (*i*NA94) that comprises 76 intracellular reactions and 148 metabolites (Fig. 1b), while the “Candidatus Hamiltonella defensa” genome has 348 metabolism genes, yielding a metabolic network (*i*NA348) with 462 reactions and 469 metabolites (Fig. 1c). To facilitate analysis of interactions between the bacterial partners and the host, a metabolic model of the host bacteriocyte was constructed to mediate the transfer of metabolites between bacterial partners and also to produce or utilize metabolites required or produced by the bacterial partners, respectively. The metabolic network of the host bacteriocyte (*i*NA332) comprises 332 genes, 236 intracellular reactions, and 253 metabolites (Fig. 1d). Finally, to generate a three-compartment model that best reflects interactions between the host and its bacteria, the three individual models were integrated into a single multicompartment model, *i*NA774, which comprises 774 genes, 774 intracellular reactions, and 550 metabolites (Fig. 1a and e). In the three-compartment model, approximately 88%, 64%, and 65% of the metabolic reactions in “Candidatus Portiera aleyrodidarum,” “Candidatus Hamiltonella defensa,” and the host carry flux, respectively (see Table S7a in the supplemental material). Additionally, ∼49% of the metabolites in the “Candidatus Portiera aleyrodidarum” *i*NA94 metabolic network are exchanged with the host. This is in sharp contrast to the 8% of the metabolite pool “Candidatus Hamiltonella defensa” *i*NA348 exchanges with the host (Fig. 1a).

**FIG 2:**
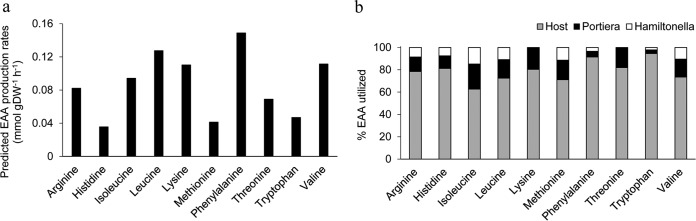
*In silico* predictions of EAA synthesis rates and utilization profiles. (a) Predictions of EAA production by “Candidatus Portiera aleyrodidarum” and host. (b) Predictions of EAA utilization profiles for host and bacteria.

The central function of the bacterial symbiosis is the provisioning of EAAs, nutrients in short supply in the host diet of plant phloem sap. Our three-compartment model generates predicted rates of production for all 10 EAAs by “Candidatus Portiera aleyrodidarum” and the host (Fig. 2a) and their utilization by each partner (Fig. 2b). The host is the principal recipient of all EAAs produced in the bacteriocyte and utilizes >60% of each EAA produced.

The models additionally provided the basis to probe the genetic robustness of the partners. We determined the proportion of metabolic genes in each genome that are essential for survival. Single gene deletions were carried out for the model representing each of the three organisms *in silico*, and the impact of gene deletions on the growth (i.e., biomass production) of each individual organism was determined. A gene was considered essential if its removal from the model resulted in a >99% decrease in growth relative to the wild type. Our simulations showed that 67%, 59%, and 9% of the genes in “Candidatus Portiera aleyrodidarum” *i*NA94, “Candidatus Hamiltonella defensa” *i*NA348, and Bemisia *i*NA332, respectively, were essential for sustaining growth *in silico* (Fig. 1f to h; see Table S8a to c in the supplemental material). These data suggest that the metabolic networks of the bacterial partners are extremely fragile while the metabolic network of the host is very robust, with multiple biochemical redundancies.

### Interactions between bacterial partners. (i) Cross-feeding of bacterial metabolic products.

**FIG 3:**
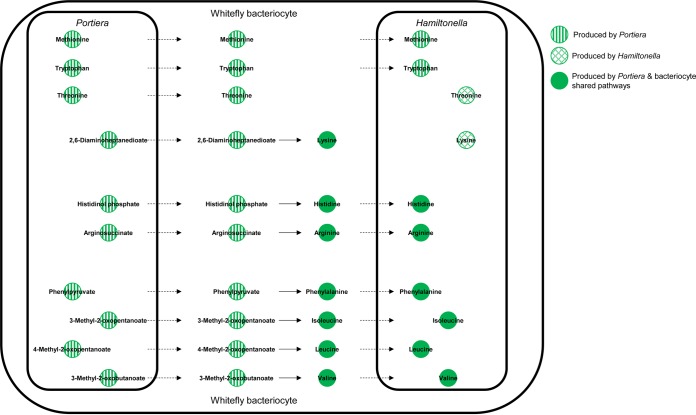
Predicted metabolic interactions between “Candidatus Portiera aleyrodidarum” and “Candidatus Hamiltonella defensa.” The dashed arrows indicate transport reactions between symbionts and hosts. The solid arrows indicate metabolite transformations occurring in the host.

To establish the extent and directionality of metabolic interactions between “Candidatus Portiera aleyrodidarum” and “Candidatus Hamiltonella defensa,” we investigated the incidence of cross-feeding between the two bacterial partners. Our simulations indicate that “Candidatus Hamiltonella defensa” is a net recipient of “Candidatus Portiera aleyrodidarum” metabolic products and that “Candidatus Portiera aleyrodidarum” receives no metabolites from “Candidatus Hamiltonella defensa” (Fig. 3). Our models reproduce the inferences from published data (11) that (i) “Candidatus Hamiltonella defensa” is auxotrophic for 8 EAAs (all except lysine and threonine); (ii) “Candidatus Portiera aleyrodidarum” overproduces tryptophan, threonine, and methionine, which are released to the host bacteriocyte, and tryptophan and methionine are consumed by “Candidatus Hamiltonella defensa”; and (iii) “Candidatus Portiera aleyrodidarum” releases precursors of the remaining 7 EAAs (arginine, histidine, isoleucine, leucine, lysine, phenylalanine, and valine), which are metabolized by the host bacteriocyte to EAAs, with all but lysine required by “Candidatus Hamiltonella defensa” (Fig. 3).

### (ii) Sharing of host-derived metabolites between bacterial partners.

**FIG 4:**
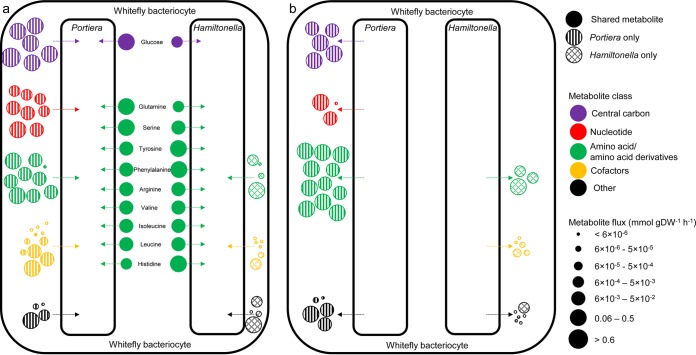
Comparison of metabolites produced and consumed by symbionts. (a) Inputs utilized by symbionts. (b) Outputs produced by symbionts. The circle colors and sizes correspond to metabolite classes and metabolite reaction flux, respectively. The metabolite class “cofactors” includes cofactors, intermediates, and side chains of cofactor biosynthesis.

The inputs derived by the two bacterial partners from the host differ in both the identities and amounts of metabolites. The metabolic models of “Candidatus Portiera aleyrodidarum” and “Candidatus Hamiltonella defensa” require 48 and 23 different inputs from the host, respectively (Fig. 1a and 4a; see Fig. S2a and b and Table S7b in the supplemental material). When considering the quantity of material that each symbiont receives as input from the host, our simulations predict “Candidatus Portiera aleyrodidarum” utilizes ∼7 times more input material (3.6 mmol g dry weight [DW]^−1^ h^−1^) from the host than “Candidatus Hamiltonella defensa” (0.5 mmol g DW^−1^ h^−1^) (see Fig. S2c and d and Table S7b in the supplemental material). Approximately 64% of the material “Candidatus Portiera aleyrodidarum” utilizes from the host is central carbon metabolism intermediates while ∼54% of “Candidatus Hamiltonella defensa” inputs are amino acids (Fig. 4a; see Fig. S2c and d in the supplemental material).

Amino acids and their derivatives dominate metabolic inputs to both bacteria, but “Candidatus Portiera aleyrodidarum” utilizes ∼20% more amino acids and their derivatives than “Candidatus Hamiltonella defensa” (see Fig. S2a and b and Table S7b in the supplemental material). Ten inputs are shared between “Candidatus Portiera aleyrodidarum” and “Candidatus Hamiltonella defensa”: the carbon source glucose, three nonessential amino acids (glutamine, serine, and tyrosine), and six EAAs (arginine, histidine, isoleucine, leucine, phenylalanine, and valine) (Fig. 4a).

We hypothesized that “Candidatus Portiera aleyrodidarum” and “Candidatus Hamiltonella defensa” may compete for the 10 shared input metabolites (Fig. 4a), resulting in depressed biomass production. The three-compartment model is unsuitable to test this hypothesis because it optimizes a single objective function (production of host amino acids) and thus cannot capture any impact of competition for metabolites on the biomass production and fitness of the bacteria. We therefore adopted a different strategy to quantify how changing the uptake fluxes of the shared metabolites affects biomass production in standalone “Candidatus Portiera aleyrodidarum” and “Candidatus Hamiltonella defensa” models. Our simulations show that, for both “Candidatus Portiera aleyrodidarum” and “Candidatus Hamiltonella defensa,” biomass production is reduced when the uptake flux of every shared metabolite is individually reduced, apart from glucose for “Candidatus Portiera aleyrodidarum” (see Fig. S3a and b in the supplemental material). The exceptional case of glucose arises because “Candidatus Portiera aleyrodidarum” does not use it for either energy production or growth; it is metabolized to 6-phosphogluconolactone (a pentose phosphate pathway intermediate), which is exported back to the host. In summary, these data point to multiple candidate instances of competition between the two bacteria for host resources, but the significance of these competitive interactions *in vivo* varies with the supply of these compounds from the host.

### Bacterial interactions with the host. (i) Bacterial metabolic outputs to the host.

We probed our three-compartment metabolic model for partitioning of metabolic functions between the bacterial partners by examining the metabolic outputs exported to the host from each bacterium. The metabolic networks of “Candidatus Portiera aleyrodidarum” and “Candidatus Hamiltonella defensa” export 24 and 14 unique metabolites to the host, respectively (Fig. 4b; see Table S7b in the supplemental material), and the compositions of these compounds differ between the two bacteria. In terms of the number of metabolites, amino acids and their derivatives dominate exports from “Candidatus Portiera aleyrodidarum,” while “Candidatus Hamiltonella defensa” releases a diverse set of metabolic products, including intermediates and side products of cofactor biosynthesis (see Fig. S4a and b and Table S7b and c in the supplemental material).

Our simulations also predict that the total quantity of metabolites exported from “Candidatus Portiera aleyrodidarum” (∼2.4 mmol g DW^−1^ h^−1^) to the host is ∼40-fold greater than from “Candidatus Hamiltonella defensa” (∼0.06 mmol g DW^−1^ h^−1^) (see Fig. S4c and d and Table S7b in the supplemental material). The two bacteria also differ in the compositions of the outputs, with amino acids and their derivatives accounting for ∼40% of the total quantity of “Candidatus Portiera aleyrodidarum” exports, while ∼93% of “Candidatus Hamiltonella defensa” outputs are central carbon metabolism intermediates (see Fig. S4c and d in the supplemental material). “Candidatus Hamiltonella defensa” has the genetic capacity to synthesize B vitamins, but these micronutrients are retained within the “Candidatus Hamiltonella defensa” cells under the conditions simulated in our model (see Table S7c in the supplemental material).

**FIG 5:**
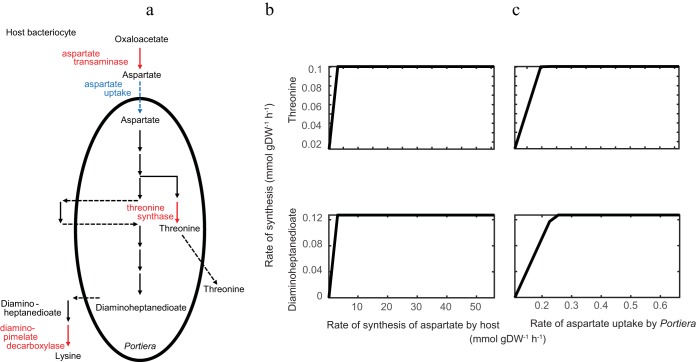
Relationship between availability of host-derived aspartate and production of the EAAs threonine and lysine in the three-compartment model. (a) Threonine and lysine synthesis from aspartate. “Candidatus Portiera aleyrodidarum” can synthesize threonine from host aspartate, but “Candidatus Portiera aleyrodidarum” and the host mediate complementary reactions in lysine biosynthesis. The metabolic reactions and transport reactions used in the simulations are shown in red and blue, respectively. (b and c) Effects of host synthesis of aspartate (b) and rate of aspartate uptake (c) by “Candidatus Portiera aleyrodidarum” on the synthesis of threonine and the lysine precursor l,l-2,6-diaminoheptanedioate by “Candidatus Portiera aleyrodidarum.” The simulation displays the reaction rates for aspartate aminotransferase (aspartate synthesis), threonine synthase (threonine synthesis), and diaminopimelate decarboxylase (lysine synthesis).

**FIG 6:**
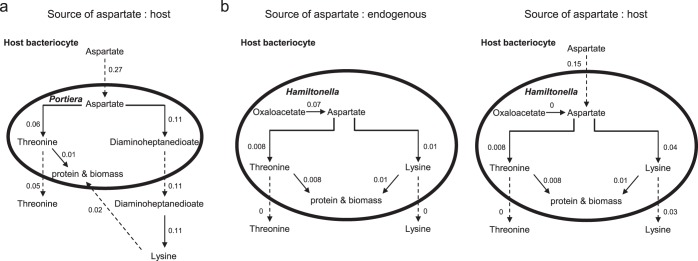
Predicted contributions of “Candidatus Portiera aleyrodidarum” and “Candidatus Hamiltonella defensa” to the supply of lysine and threonine to the host. The reaction rates (solid arrows) and transport rates (dashed arrows) in the three-compartment model (millimoles gram DW^−1^ hour^−1^) are shown. (a) “Candidatus Portiera aleyrodidarum”-mediated production of threonine and the lysine precursor l,l-2,6-diaminoheptanedioate from host aspartate (“Candidatus Portiera aleyrodidarum” lacks the genetic capacity to synthesize aspartate). (b) “Candidatus Hamiltonella defensa”-mediated production of threonine and lysine synthesis from endogenously generated aspartate (left) and host aspartate (right).

Despite having redundant metabolic pathways (i.e., encoded by both bacteria) for threonine and lysine biosynthesis, our simulations indicate “Candidatus Portiera aleyrodidarum” alone supplies threonine and 2,6-diaminoheptanedioate (a lysine precursor) to the host. The amounts of these compounds released by “Candidatus Portiera aleyrodidarum” are proportional to the aspartate supply from the host under physiologically meaningful conditions (Fig. 5). (The saturation of threonine and 2,6-diaminoheptanediote production by “Candidatus Portiera aleyrodidarum” at high aspartate supply [Fig. 5] is an artifact that arises when the production of these compounds exceeds the amount that supports the maximal host growth rate permitted in the model.) Additionally, the host supplies all the aspartate that “Candidatus Portiera aleyrodidarum” uses for their synthesis (Fig. 6a; see Table S7a in the supplemental material). Taken together, these findings strongly suggest that production of these EAAs is regulated by the substrate supply from the host. Our model predicts similar linear substrate-product relationships for the other 8 EAAs (see Fig. S5 in the supplemental material).

Complementary analyses investigated the metabolic conditions in the host bacteriocytes that may stimulate “Candidatus Hamiltonella defensa” to release lysine and threonine. “Candidatus Hamiltonella defensa” has the genetic capacity to synthesize aspartate, and none of the lysine and threonine synthesized from this endogenous aspartate is released (Fig. 6b). However, when “Candidatus Hamiltonella defensa” is simulated to receive aspartate from the host, it exports ∼30% of the total lysine needed by the host and “Candidatus Portiera aleyrodidarum” but exports no threonine (Fig. 6b). These data suggest that “Candidatus Hamiltonella defensa” makes little or no contribution to the EAA requirements of the host under the metabolic conditions captured by our models.

### (ii) Roles of “Candidatus Portiera aleyrodidarum” and “Candidatus Hamiltonella defensa” in recycling host nitrogen.

**FIG 7:**
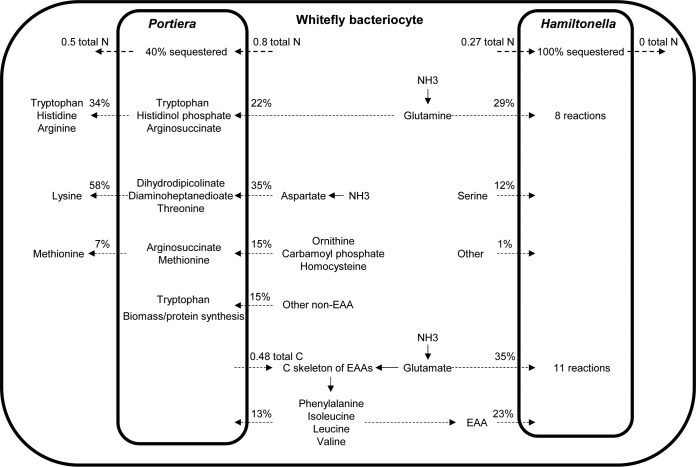
Bemisia-symbiont-mediated ammonia assimilation and nitrogen recycling. The inferred fluxes for total nitrogen assimilated and released by symbionts were measured in millimoles gram DW^−1^ hour^−1^. The dashed arrows represent transport fluxes between hosts and symbionts. The reaction rates are shown as percentages of the total nitrogen entering or leaving the symbiont cell. The solid arrows represent host-mediated reactions.

To determine if “Candidatus Portiera aleyrodidarum” and “Candidatus Hamiltonella defensa” mediate recycling of host nitrogen (here referred to as N), we tracked the fates of ammonia (a nitrogenous waste product of the host), nonessential amino acids, and amino acid derivatives generated in the host bacteriocyte. Our simulations indicate that ∼40% the total flux of N into “Candidatus Portiera aleyrodidarum” is sequestered. Approximately 0.8 mmol g DW^−1^ h^−1^ of N-containing metabolites is received by “Candidatus Portiera aleyrodidarum” from the host and 0.5 mmol g DW^−1^ h^−1^ of N-rich metabolites, mostly in a form the host cannot synthesize, is released back to the host (Fig. 7).

The dominant classes of N metabolites “Candidatus Portiera aleyrodidarum” receives as input are aspartate (35% of total N); glutamine (22%); other nonessential amino acids (15%); ornithine, carbamoyl phosphate, and homocysteine (15%); and essential amino acids (13%). Aspartate is used by “Candidatus Portiera aleyrodidarum” to synthesize threonine and the lysine precursors 2,3-dihydrodipicolinate and 2,6-diaminoheptanedioate. These three metabolites constitute 58% of the total N released by “Candidatus Portiera aleyrodidarum” to the host. Glutamine is used in three EAA-biosynthetic pathways to make tryptophan, histidinol phosphate (a histidine precursor), and arginosuccinate (an arginine precursor), together accounting for 34% of the total N output. Ornithine, carbamoyl phosphate, and homocysteine are used to make arginosuccinate and methionine, representing 7% of the total N output (Fig. 7).

In addition to recycling N, “Candidatus Portiera aleyrodidarum” produces 0.48 mmol g DW^−1^ h^−1^ of the C skeletons of the EAAs isoleucine, leucine, phenylalanine, and valine. These C skeletons are exported to the host bacteriocyte, where the terminal transaminase reaction mediating their synthesis occurs. A proportion of these essential amino acids is then imported back into “Candidatus Portiera aleyrodidarum” for protein synthesis and biomass production (Fig. 4 and 7).

The total flux of N received by “Candidatus Hamiltonella defensa” from the host is 0.27 mmol g DW^−1^ h^−1^, all of which is sequestered by the bacterium. The dominant classes of N metabolites “Candidatus Hamiltonella defensa” receives as input are glutamate (35%), glutamine (29%), serine (12%), and essential amino acids (23%) (Fig. 7).

### (iii) Net cost or benefit of bacterial symbionts to the host.

To investigate the metabolic significance of the two bacterial partners to the host, host growth was compared between the three-compartment model and two-compartment models generated by omitting all reactions associated with one bacterium. In the two-compartment models, uptake fluxes for glucose, fructose, ammonium, and phosphate, the main sources of C, N, and P were capped at the observed uptake rates in the three-compartment model. Our simulations indicated that a two-compartment “Candidatus Hamiltonella defensa”-host model was incapable of supporting host growth. In contrast, removal of “Candidatus Hamiltonella defensa” resulted in an ∼12% increase in the host growth rate (see Table S7d in the supplemental material), together with 11, 6, and 15% increases in the uptake rates of the shared metabolites glucose, glutamine, and serine, respectively. The uptake rates of the EAAs with host-mediated terminal reactions (arginine, histidine, isoleucine, leucine, phenylalanine, and valine) remained unchanged, but the export of tryptophan, threonine, and most EAA precursors from “Candidatus Portiera aleyrodidarum” increased by 0.5 to 22%. Exceptionally, the efflux of methionine and the C skeletons of the essential amino acids isoleucine, leucine, and valine were reduced by 2 to 18% relative to the three-compartment model including “Candidatus Hamiltonella defensa” (see Table S7d in the supplemental material). Altogether, these data highlight the impact “Candidatus Hamiltonella defensa” has on nutrient exchange in this three-partner symbiosis and provide data on the nutritional costs associated with harboring “Candidatus Hamiltonella defensa” by the host.

## DISCUSSION

Substantial insights into intracellular bacterial symbionts with much-reduced genomes have been gained from the sequencing of their genomes and especially analysis of their complement of metabolism genes (5, 8). Genome-informed metabolic modeling, as used in this study, can further enhance our understanding of the metabolic functions of these bacteria by providing quantitative predictions of metabolic flux both within individual bacteria and between the partners in an association. In particular, our modeling of the three-partner symbiosis between the bacteria “Candidatus Portiera aleyrodidarum” and “Candidatus Hamiltonella defensa” with the whitefly host highlights the distinctive traits of bacterial symbionts with small genomes. Notably, “Candidatus Portiera aleyrodidarum” is highly cooperative, with >60% of essential amino acids synthesized in the system predicted to be allocated to the host (Fig. 2); these values are appreciably greater than the predicted 22 to 50% transfer of essential amino acids to the host in the aphid-Buchnera symbiosis (28). On the other hand, the susceptibility of metabolic networks of both the bacterial symbionts in the whitefly host to genetic perturbation parallels the previously described fragility of the Buchnera metabolic network (28) and contrasts sharply with the robustness of the metabolic networks in free-living bacteria, such as E. coli, with 15% essential genes (*i*JO1366) (27), as well as the metabolic network of the whitefly host cell, with 9% essential genes (Fig. 1). The fragility of the metabolic networks of these bacterial symbionts reflects their low metabolic redundancy, arising from the evolutionary loss of many metabolism-related genes through relaxed selection and genomic deterioration in the host habitat.

In this discussion, we consider these interactions first from the perspective of metabolic inputs to the bacteria and then from the perspective of metabolic outputs from the bacteria to the host.

### Metabolic inputs to the bacteria.

The inputs to symbiotic bacteria mostly represent metabolic costs for the host. In a multipartner association, these costs can be compounded by competition between symbionts for the same host metabolites (29). Our simulations indicate that “Candidatus Portiera aleyrodidarum” and “Candidatus Hamiltonella defensa” share 10 host-supplied metabolites and that competition for these shared resources decreases bacterial growth rates and may impact the capacity of each symbiont to provide enough nutrients for the host, thereby negatively impacting host fitness. Competition can incur additional costs by driving the evolution or expression of antagonistic traits in the bacteria (29), but under some circumstances, it can stabilize microbial communities (30). In the whitefly symbiosis, competition may be tempered by dependence; specifically, “Candidatus Hamiltonella defensa” is a net recipient of “Candidatus Portiera aleyrodidarum” metabolic products, some of which are essential for the growth of “Candidatus Hamiltonella defensa.” It is also possible that the host suppresses competition by isolating every cell of both symbionts within an individual host membrane, known as the symbiosomal membrane, which may contribute to the regulation of metabolite flux (31, 32).

It has been suggested for many animal-microbial symbioses that some inputs to the microbial symbionts are metabolic waste products of the host, and consequently, their utilization by the symbiont is cost free for the host and potentially advantageous by reducing the energy costs of eliminating the waste metabolite (33). When these inputs are metabolized by the symbiont to a product valuable to the host, i.e., metabolic recycling (especially in relation to nitrogenous waste), the net benefit to the host is enhanced further. Definitive conclusions as to the role of bacterial symbionts in the whitefly symbiosis in removal of nitrogenous waste and nitrogen recycling must await identification of the insect's nitrogenous waste products. However, as in aphids, a related insect group, the dominant nitrogen waste product of whiteflies is likely ammonia (34), and the extensive predicted assimilation of ammonia into nonessential amino acids delivered to both “Candidatus Portiera aleyrodidarum” and “Candidatus Hamiltonella defensa” is indicative of a role of these bacteria as sinks for host waste nitrogen. Our analyses suggest that this potential benefit to the host is compounded by the recycling of more than half of the nitrogenous input to EAAs in “Candidatus Portiera aleyrodidarum” (but not “Candidatus Hamiltonella defensa”). This level of recycling is high compared to the aphid-Buchnera symbiosis, where the predicted nitrogen input is 11-fold greater than EAA release (35). The basis for this difference is the substantially smaller genome size and metabolic scope of “Candidatus Portiera aleyrodidarum” and “Candidatus Portiera aleyrodidarum” than of Buchnera, so that “Candidatus Portiera aleyrodidarum” requires a smaller amount and diversity of nitrogenous metabolites to support its metabolic network and growth. The implication that EAA-releasing bacterial symbionts with smaller genome sizes may have smaller demands for limiting host nitrogen, making them less costly to the host, could provide a previously unconsidered selection pressure for reduced genome size. This prediction can be tested by analysis of additional symbiotic bacteria with different genome sizes.

### Metabolic outputs from the bacterial symbionts to the host.

Our models indicate that the host requirement for EAAs can be met quantitatively by the primary symbiont, “Candidatus Portiera aleyrodidarum.” Although “Candidatus Hamiltonella defensa” is capable of synthesizing two EAAs, lysine and threonine (24, 36), its metabolic network is structured to synthesize sufficient lysine and threonine for its own growth requirements and not for overproduction to support the host nutritional requirements. This result illustrates how certain metabolic traits cannot be predicted from the gene content of a bacterium and are driven by the architecture of the metabolic network. The evidence from this study that “Candidatus Hamiltonella defensa” is a nutritional parasite that contributes little or nothing to the EAA or B vitamin nutrition of its host contrasts with the genomic and experimental data showing a nutritional role of all bacterial taxa in multipartner symbioses in some other insect groups (5, 8), although the metabolic networks have not been investigated systematically in most systems.

Our analyses indicate that the source of the precursor, aspartate, plays a critical role in shaping the quantitative differences between “Candidatus Portiera aleyrodidarum” and “Candidatus Hamiltonella defensa” with respect to lysine and threonine production. The finding that all the aspartate used by “Candidatus Portiera aleyrodidarum” is derived from the host and that lysine and threonine are synthesized in proportion to the aspartate supply with no network constraints parallels empirical evidence that EAA production by a different symbiosis, Buchnera in aphids, is regulated by the substrate supply (37). In contrast, the “Candidatus Hamiltonella defensa” network is structured to synthesize enough aspartate to meet its biosynthetic needs, and hence, the bacterium is predicted not to overproduce and release lysine and threonine (Fig. 6). Even when provided with an unlimited supply of exogenous aspartate, the metabolic network of “Candidatus Hamiltonella defensa” is predicted to contribute only a small fraction of the total lysine required by the host and to release no threonine (Fig. 6).

Our interpretation that “Candidatus Hamiltonella defensa” does not contribute substantially to the EAA nutrition of the host is broadly consistent with the pattern that most instances of EAA biosynthesis partitioned to a secondary symbiont involve EAAs that are energetically expensive to produce, e.g., histidine, methionine, and tryptophan (8). The EAAs that “Candidatus Hamiltonella defensa” can synthesize are not energetically costly, and a contribution of secondary symbionts to their synthesis is either unknown (threonine) or apparently uncommon (lysine).

### Concluding comments.

This first metabolic model of a multipartner symbiosis in an insect host offers quantitative predictions about the functions of two bacterial symbionts sharing a single host cell. The key experimental advantage is that the models create precise hypotheses for empirical testing. For systems such as whiteflies, where experimental studies are technically demanding and time-consuming because of the very small size of the insects, this *in silico* study fast tracks discovery. As metabolic models come to be applied to other associations, it will become feasible to conduct detailed comparisons, with the opportunity to investigate the roles of host and symbiont traits that define the convergent functions of EAA overproduction and release, as well as important differences that may be related to the evolutionary histories of the partners and the ecological circumstance of the different symbioses.

## MATERIALS AND METHODS

### Metabolic-model reconstruction for individual species.

To generate genome scale metabolic models of “Candidatus Portiera aleyrodidarum” (*i*NA94) (see Table S1 in the supplemental material) and “Candidatus Hamiltonella defensa” (*i*NA348) (see Table S2 in the supplemental material) associated with B. tabaci, reciprocal BLAST searches of symbiont genomes against E. coli strain K-12 substrain MG1655 were used to identify gene orthologs. The criteria for the bidirectional matches included in the model were as follows: an E value of <1e−5, 35% amino acid sequence identity, and match lengths of >70% of the lengths of both query and subject. The list of orthologous genes generated from reciprocal BLAST searches was compared to that of the E. coli strain K-12 substrain MG1655 metabolic model, *i*JO1366 (27), and reactions encoded by these genes were manually extracted to create a draft model. Organism-specific features and genes encoding metabolic reactions absent in *i*JO1366 were added to the draft model after extensive literature review and searches of the BioCyc, KEGG, EcoCyc, BiGG, and BRENDA databases (38–41). Genes annotated as pseudogenes and their associated reactions were excluded from “Candidatus Portiera aleyrodidarum” and “Candidatus Hamiltonella defensa” model reconstructions. To generate a metabolic model of B. tabaci (*i*NA332) (see Table S3 in the supplemental material), reactions capable of generating or consuming dead-end metabolites in each bacterial-symbiont model were identified and incorporated into a draft reconstruction of the host bacteriocyte metabolic network. Additional host reactions completing metabolic pathways for each added reaction and host gene products enriched in host bacteriocytes were incorporated into a final reconstruction of the metabolic network. With the exception of the objective function, exchange, and demand reactions, all reactions included in each model were mass and charge balanced. All the metabolic networks were visualized using Cytoscape_v3.4.0 (42).

### Reconstruction of the integrated three-partner metabolic model.

To integrate the three individual models (“Candidatus Portiera aleyrodidarum” *i*NA94, “Candidatus Hamiltonella defensa” *i*NA348, and Bemisia bacteriocyte *i*NA332) into a single multicompartment model, reactions and metabolites assigned to each partner were renamed, with reactions in POR (“Candidatus Portiera aleyrodidarum”), HAM (“Candidatus Hamiltonella defensa”), and BTA (Bemisia) and metabolites in [p] (“Candidatus Portiera aleyrodidarum”), [h] (“Candidatus Hamiltonella defensa”), and [b] (Bemisia). The stoichiometric matrices of the three models were combined to generate an integrated model, *i*NA774 (see Table S4 in the supplemental material). Transport reactions were added to connect the compartments of “Candidatus Portiera aleyrodidarum” and “Candidatus Hamiltonella defensa” to the Bemisia compartment, which was adopted as the site for the exchange of metabolites with the external environment.

Transport reactions for each bacterial compartment were selected based on the following criteria: (i) dead-end metabolites in each bacterial compartment were assigned transport reactions to or from the host bacteriocyte to allow enzymatic reactions mediating their synthesis or consumption to carry flux and (ii) metabolites produced in one species compartment for which another species compartment was auxotrophic were assigned transport reactions to and from the host bacteriocyte. The COBRA toolbox (43), run in Matlab (MathWorks Inc., Natick, MA), was used for model testing and determining metabolic-flux distributions in each model using the Gurobi solver (44).

### Metabolic-model medium composition and reaction constraints.

All model simulations were performed under aerobic conditions with a maximum oxygen uptake rate of 20 mmol g DW^−1^ h^−1^. In the absence of empirical data on the compositions and concentrations of metabolites in the external medium bathing the host bacteriocyte (insect hemolymph), we adopted the most parsimonious strategy of a minimal medium comprising glucose and fructose as carbon sources, ammonia and homocysteine as sources of nitrogen, and hydrogen sulfide as a source of sulfur. Cofactors added to the model medium were thiamine monophosphate, thiamine diphosphate, riboflavin, pyridoxine 5-phosphate, and nicotinate d-ribonucleotide. Water, protons, carbon dioxide, iron(II), iron(III), and phosphate were allowed to freely diffuse across the bacteriocyte cell membrane. The maximum uptake fluxes of all medium components were capped at 100 mmol g DW^−1^ h^−1^. The upper bounds of all whitefly intracellular reactions were constrained by the transcript abundances of their respective associated genes, with the exception of asparagine synthase, diaminopimelate decarboxylase, argininosuccinate lyase, histidinol dehydrogenase, phenylalanine hydroxylase, phosphoserine phosphatase, and valine transaminase, which were constrained using the absolute values of amino acid concentrations measured *in vivo*. Empirically determined concentrations of amino acids were also used to constrain two “Candidatus Portiera aleyrodidarum” reactions, methionine synthase (METS) and threonine synthase (THRS) (see Table S4 in the supplemental material). The transcriptome (RNA-Seq) data used to constrain the upper bounds of all B. tabaci reactions in the metabolic model were taken from a previous study (11). The lower bounds of the biomass reactions of “Candidatus Portiera aleyrodidarum” and “Candidatus Hamiltonella defensa” were constrained based on their relative abundance data inferred from empirical data obtained in this study (see Table S5 in the supplemental material).

### Objective function.

A single objective function representing the total amino acid content in the whole body of an adult whitefly was optimized for all our three-compartment (*i*NA774) model simulations. We selected this community objective function because the primary function of this bacterial-host symbiosis is the provisioning of EAAs that supplement the EAA-deficient plant sap diet of the insect host (see the introduction). Additionally, biomass reactions for “Candidatus Portiera aleyrodidarum” and “Candidatus Hamiltonella defensa” were included in each bacterial compartment to represent bacterial growth. The coefficients for the components in the biomass reaction of the bacterial models (“Candidatus Portiera aleyrodidarum” *i*NA94 and “Candidatus Hamiltonella defensa” *i*NA348) (see Table S6a in the supplemental material) were derived from the biomass equation of the metabolic model for another bacteriocyte symbiont, *Buchnera i*SM199, generated using the experimental data from reference 35. “Candidatus Portiera aleyrodidarum,” “Candidatus Hamiltonella defensa,” and Buchnera are all gammaproteobacteria with highly reduced genomes and are endosymbionts in insects of the same suborder, Sternorryncha. The following modifications were made to the Buchnera biomass equation to generate biomass equations for “Candidatus Portiera aleyrodidarum” and “Candidatus Hamiltonella defensa”: (i) the biomass coefficients for each amino acid were recalculated to reflect a “selfish” model where EAAs are produced solely to support bacterial protein synthesis and biomass increase, (ii) cofactors and soluble pool metabolites absent from the Buchnera model but present in the “Candidatus Portiera aleyrodidarum” and “Candidatus Hamiltonella defensa” models were given small coefficients (0.00005) to ensure their production by each model, and (iii) H_2_O was included as a substrate and protons as products in the ATP maintenance equations (45).

For B. tabaci *i*NA332, the objective function was defined as the total abundance of each amino acid in the host protein and was quantified as follows (see Table S6a in the supplemental material). First, every transcript in the whole-body transcriptome of an adult whitefly was translated *in silico*; then, the number of residues of each amino acid in each protein was summed and multiplied by the estimated abundance of the transcript in the transcriptome. The estimated abundance of each transcript was determined by considering the lowest-abundance transcript as present once and normalizing the count for the more abundant transcripts accordingly (i.e., the abundance of each transcript was divided by the abundance of the least abundant transcript in the transcriptome) (see Table S6b in the supplemental material). Transcripts with zero abundance were considered to be present once to account for low-abundance transcripts omitted from the transcriptome during sample preparation and processing. Assuming a cellular protein content of 70.6% (46), the fractional contribution of each amino acid to the predicted proteome was used to calculate the amino acid coefficients for the B. tabaci biomass reaction (see Table S6c in the supplemental material), as described in reference 45. ATP maintenance requirements for the synthesis and replication of each amino acid were calculated by following the protocol of Thiele and Palsson (45).

### Metabolite analysis.

The free amino acid content of bacteriocytes from B. tabaci MEAM1 (mitochondrial cytochrome *c* oxidase subunit 1 [mtCO1]; GenBank accession no. KM507785) (11) was quantified to provide constraints for 9 reactions (see Table S4a in the supplemental material). Briefly, multiple female whiteflies were dissected with fine pins (11) to generate a pool of ∼2,400 bacteriocytes for each of nine biological replicates. All the samples were hand homogenized in phosphate-buffered saline (PBS) on ice. The homogenates were centrifuged at 18,000 × *g* for 5 min at 4°C, and the supernatants, mixed with an equal volume of 40 mM HCl, were incubated on ice for 30 min and centrifuged at 18,000 × *g* for 15 min at 4°C. An aliquot of the supernatant (20 μl) was frozen for subsequent protein quantification. The remaining supernatant was filtered through a 0.45-μm filter plate (Millipore) by centrifugation at 1,500 × *g* for 10 min. Then, 2.5 μl filtrate was derivatized with AccQ Tag (Waters), following the manufacturer's protocol, and injected into a Waters Acquity ultraperformance liquid chromatograph (UPLC) with a photodiode array (PDA) detector and an AccQ-Tag Ultra 2.1- by 100-mm column. The gradient used was as follows: 0 to 0.54 min, 99.9% A and 0.1% B; 0.54 to 5.74 min, 90.9% A and 9.1% B; 5.74 to 7.74 min, 78.8% A and 21.2% B; 7.74 to 8.04 min, 40.4% A and 59.6% B; 8.04 to 8.64 min, 10% A and 90% B; 8.05 to 8.64 min, 10% A and 90% B; 8.64 to 8.73 min, 99.9% A and 0.1% B; 8.73 to 9.50 min, 99.9% A and 0.1% B (linear between time points), where A is 90% AccQ-*Taq* Ultra Eluent A in water and B is Accq-*Taq* Ultra Eluent B. Amino acids were determined by comparing their retention times with standards at 1, 5, 10, 50, and 100 pmol ammonia and protein-amino acids μl^−1^ (Waters amino acid hydrolysate standard 088122 supplemented with asparagine, tryptophan, and glutamine) and quantified with standard curves. The amino acids were normalized to the protein content determined by DC protein assay (Bio-Rad) with 0 to 10 mg bovine serum albumin ml^−1^ as a standard.

## Supplementary Material

Supplemental material
