# Supplementary material for: Cooperative Metabolism in a Three-Partner Insect-Bacterial Symbiosis Revealed by Metabolic Modeling
Source: J Bacteriol. 2017 Jul 11;199(15):e00872-16. doi: 10.1128/JB.00872-16 (PMC5512215; doi:10.1128/JB.00872-16)
Supplement: Supplemental material [file supp_199_15_e00872-16__index.html]

Supplemental material 

# Cooperative Metabolism in a Three-Partner Insect-Bacterial Symbiosis Revealed by Metabolic Modeling

## Supplemental material

- Supplemental file 1 -

  Fig. S1 (Overview of *Portiera* and *Hamiltonella* genomes), S2 and S4 (Metabolic inputs to [S2] and outputs from [S4] bacterial compartments of 3-species integrated metabolic model), S3 (Concentrations of shared metabolites and biomass production), S5 (Precursor availability and essential amino acid production), and S6 (Cooperative pantothenate synthesis involving shared pathways between *Bemisia* and *Portiera*) and legends to Tables S1 to S8

  PDF, 6.2M
- Supplemental file 2 -

  Table S1 (Stand-alone *Portiera* metabolic model)

  XLS, 154K
- Supplemental file 3 -

  Table S2 (Stand-alone *Hamiltonella* metabolic model)

  XLS, 333K
- Supplemental file 4 -

  Table S3 (Stand-alone *Bemisia* metabolic model)

  XLS, 210K
- Supplemental file 5 -

  Table S4 (Three-compartment *Portiera-Hamiltonella-Bemisia* model)

  XLS, 525K
- Supplemental file 6 -

  Table S5 (Relative densities of *Portiera* and *Hamiltonella*)

  XLSX, 12K
- Supplemental file 7 -

  Table S6 (Objective function components in metabolic models and amino acid proportions)

  XLS, 8.9M
- Supplemental file 8 -

  Table S7 (Single gene deletions for stand-alone metabolic models)

  XLS, 376K
- Supplemental file 9 -

  Table S8 (Three-compartment metabolic model predicted reaction fluxes and inputs, outputs, and predicted fluxes)

  XLSX, 35K
