# Supplementary material for: Cooperative Metabolism in a Three-Partner Insect-Bacterial Symbiosis Revealed by Metabolic Modeling
Source: J Bacteriol. 2017 Jul 11;199(15):e00872-16. doi: 10.1128/JB.00872-16 (PMC5512215; doi:10.1128/JB.00872-16)
Supplement: Supplemental material [file JB.00872-16_zjb999094413s1.pdf]

Supplementary Material

Supplementary figures

Figure S1. Overview of *Portiera* and *Hamiltonella* genomes. Multi-level pie chart shows summary statistics for symbiont genomes. Genes included in the construction of metabolic models are shown in blue. Numbers and percentages in parentheses indicate the number of genes in each subgroup and their percentage abundance relative to all ORFs in the symbiont genomes.

Figure S2. Metabolic inputs to bacterial compartments of 3-species integrated metabolic model Percentage composition of metabolite classes based on metabolite counts for **a)** *Portiera* and **b)** *Hamiltonella* and on metabolite fluxes for **c)** *Portiera* and **d)** *Hamiltonella.* Metabolite counts for each compound class are shown in parenthesis. Total metabolite count and fluxes into each bacterium are indicated on top of each pie chart. Details of individual metabolite count and fluxes are provided in Table S7b. *Metabolite class includes all cofactors, intermediates and side products of cofactor biosynthesis.

Figure S3. Effect of varying concentrations of shared metabolites on a) *Portiera* and b) *Hamiltonella* biomass production.

Figure S4. Metabolic outputs from bacterial compartments of 3-species integrated metabolic model Percentage composition of output metabolite classes based on metabolite counts for **a)** *Portiera* and **b)** *Hamiltonella* and metabolite fluxes for **c)** *Portiera* and **d)** *Hamiltonella.*

Metabolite counts for each compound class are shown in parenthesis. Total metabolite count and fluxes out of each bacterium are indicated on top of each pie chart. Details of individual metabolite count and fluxes are provided in Table S7b. *Metabolite class includes all cofactors, intermediates and side products of cofactor biosynthesis.

Figure S5. Effect of host precursor synthesis and precursor uptake rate by *Portiera* on the synthesis of EAAs.

Supplementary tables

Table S1. Stand-alone *Portiera* metabolic model **a)** reaction list and **b)** metabolite list.

Table S2. Stand-alone *Hamiltonella* metabolic model **a)** reaction list and **b)** metabolite list.

Table S3. Stand-alone *Bemisia* metabolic model **a)** reaction list and **b)** metabolite list.

Table S4. Three-compartment *Portiera-Hamiltonella-Bemisia* model **a)** reaction list and **b)** metabolite list.

Table S5. Relative density of *Portiera* and *Hamiltonella* quantified as 16S rRNA gene amplicon abundance relative to the *B. tabaci β*-actin gene. Symbionts were enumerated by qPCR using 16S primers specific for *Portiera* (Port73-F [GTGGGGAATAACGTACGG], Port266-R [CTCAGTCCCAGTGTGGCTG]) (46) and *Hamiltonella* (H-16S-Fis [GCATCGAGTGAGCACAGTTT], H-16S-Ris [TATCCTCTCAGACCCGCTAGA]) (18), and normalized to the *B. tabaci* β-actin gene (primers Actin-F [TCTTCCAGCCATCCTTCTTG], Actin-R [CGGTGATTTCCTTCTGCATT]) (47). DNA was extracted from three replicates of 20 pooled whitefly adults at 7 d post emergence using DNeasy Blood & Tissue Kit (QIAGEN) according to the manufacturer's protocol. All symbiont qPCR were performed using the CFX96 ™ Real-Time PCR Detection System (Bio-Rad) with SYBR-Green detection (iQTM SYBER@ Green Supermix, Bio-Rad). The amplification programs for each symbiont primer set are as

follows: 95 °C for 3 min, followed by 40 cycles of: 95 °C for 15 s and 60 °C for 30 s. Melt curves were generated after each assay to verify the specificity of the amplification. Standard curves were constructed and amplification efficiency (E) was estimated as previously described (1). The relative symbiont density was calculated using the $2^{-\Delta Ct}$ method (2).

Table S6. a) Objective function components in *Portiera, Hamiltonella*, and *Bemisia* metabolic models. **b)** Estimation of amino acid proportion in *Bemisia* protein. **c)** Calculation of amino acid stoichiometry for *Bemisia* model. **d)** Estimation of amino acid proportion in *Buchnera*. **e)** Calculation of amino acid stoichiometry for 'selfish' *Buchnera* model.

Table S7. a) Three-compartment *Portiera-Hamiltonella-Bemisia* metabolic model predicted reaction fluxes. **b)** List of inputs and outputs and their predicted fluxes for bacterial partners (*Portiera* and *Hamiltonella*) in three compartment model. **c)** List of cofactors, intermediates and side products produced/exported from *Hamiltonella* cofactor biosynthetic pathways. **d)** Comparison of predicted reaction fluxes for three-compartment (*Portiera-Hamiltonella-Bemisia*) and two-compartment (*Portiera-Bemisia*) metabolic models.

Table S8. Single gene deletions for stand-alone **a)** *Portiera,* **b)** *Hamiltonella* and **c)** *Bemisia* metabolic models.

References

1. Luan J-B, Chen W, Hasegawa DK, Simmons AM, Wintermantel WM, Ling K-S, Fei Z, Liu S-S, Douglas AE. 2015. Metabolic coevolution in the bacterial symbiosis of whiteflies and related plant sap-feeding insects. Genome biology and evolution 7:2635-2647.

2. Schmittgen TD, Livak KJ. 2008. Analyzing real-time PCR data by the comparative CT method. Nature protocols 3:1101-1108.

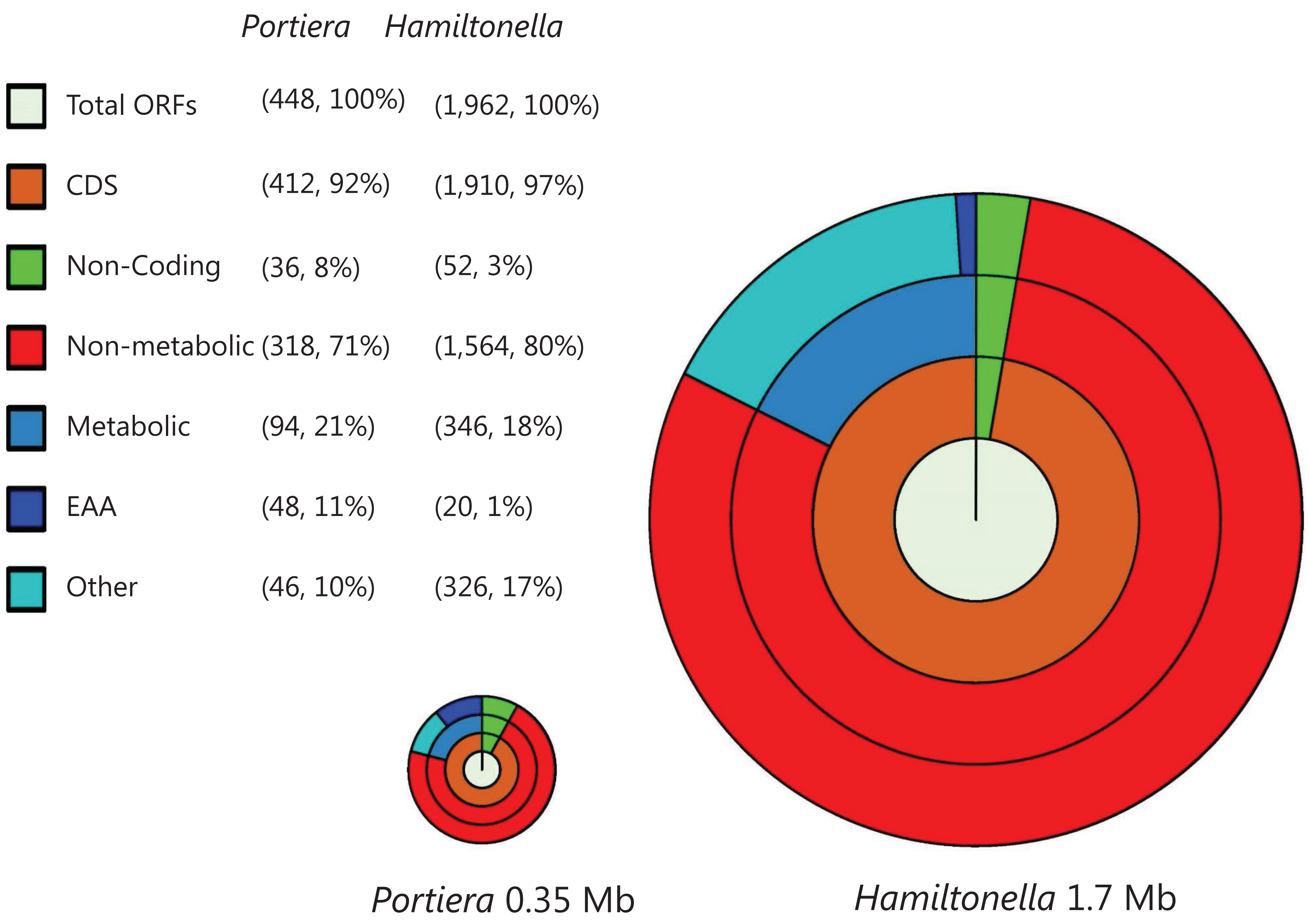

Figure S1. Overview of *Portiera* and *Hamiltonella* genomes. Multi-level pie chart shows summary statistics for symbiont genomes. Genes included in the construction of metabolic models are shown in blue. Numbers and percentages in parentheses indicate the number of genes in each subgroup and their percentage abundance relative to all ORFs in the symbiont genomes

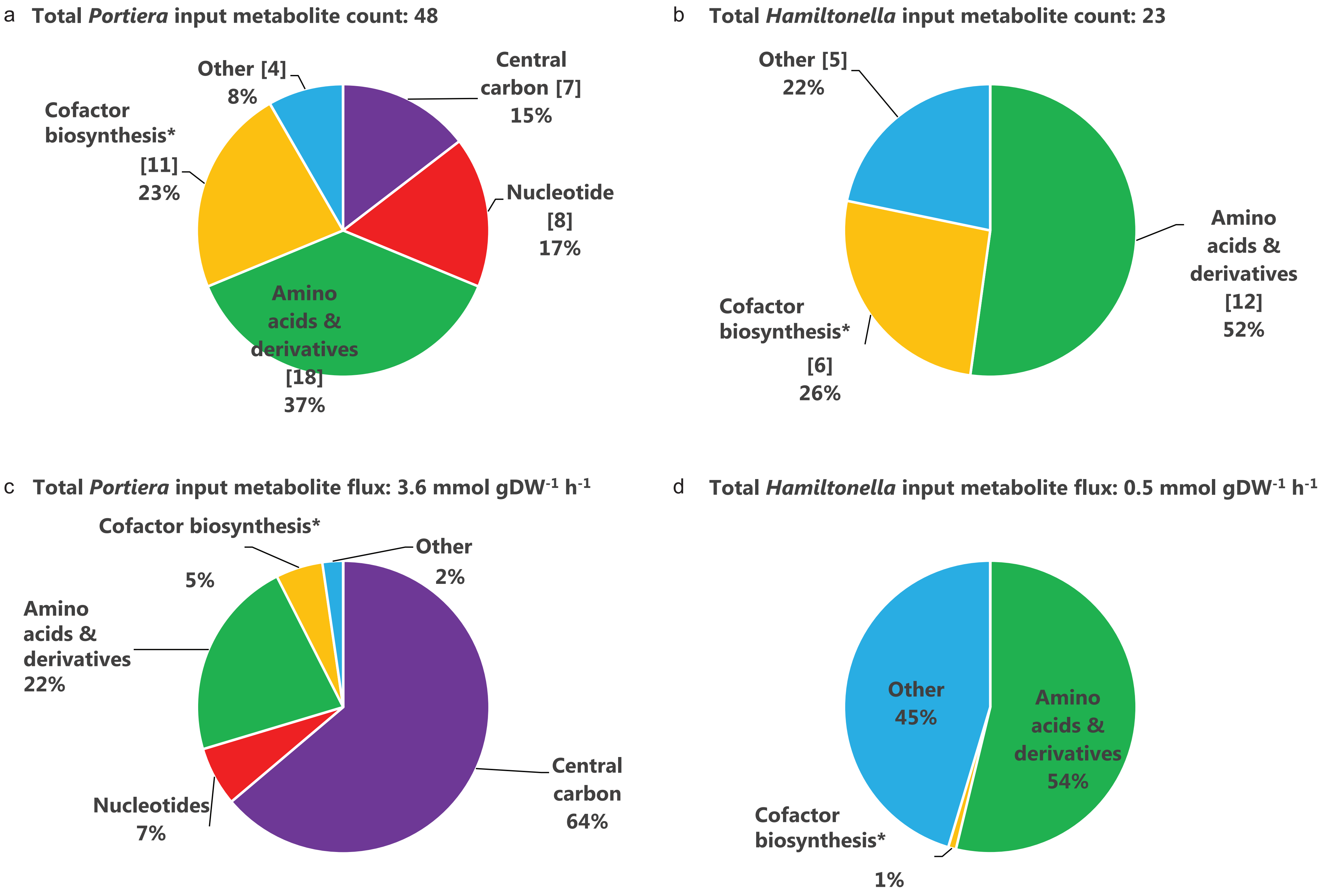

Figure S2. Metabolic inputs to bacterial compartments of 3-species integrated metabolic model Percentage composition of metabolite classes based on metabolite counts for a) *Portiera* and b) *Hamiltonella* and on metabolite fluxes for c) *Portiera* and d) *Hamiltonella*. Metabolite counts for each compound class are shown in parenthesis. Total metabolite count and fluxes into each bacterium are indicated on top of each pie chart. Details of individual metabolite count and fluxes are provided in Table S7b. *Metabolite class includes cofactors, intermediates and side products of cofactor biosynthesis.

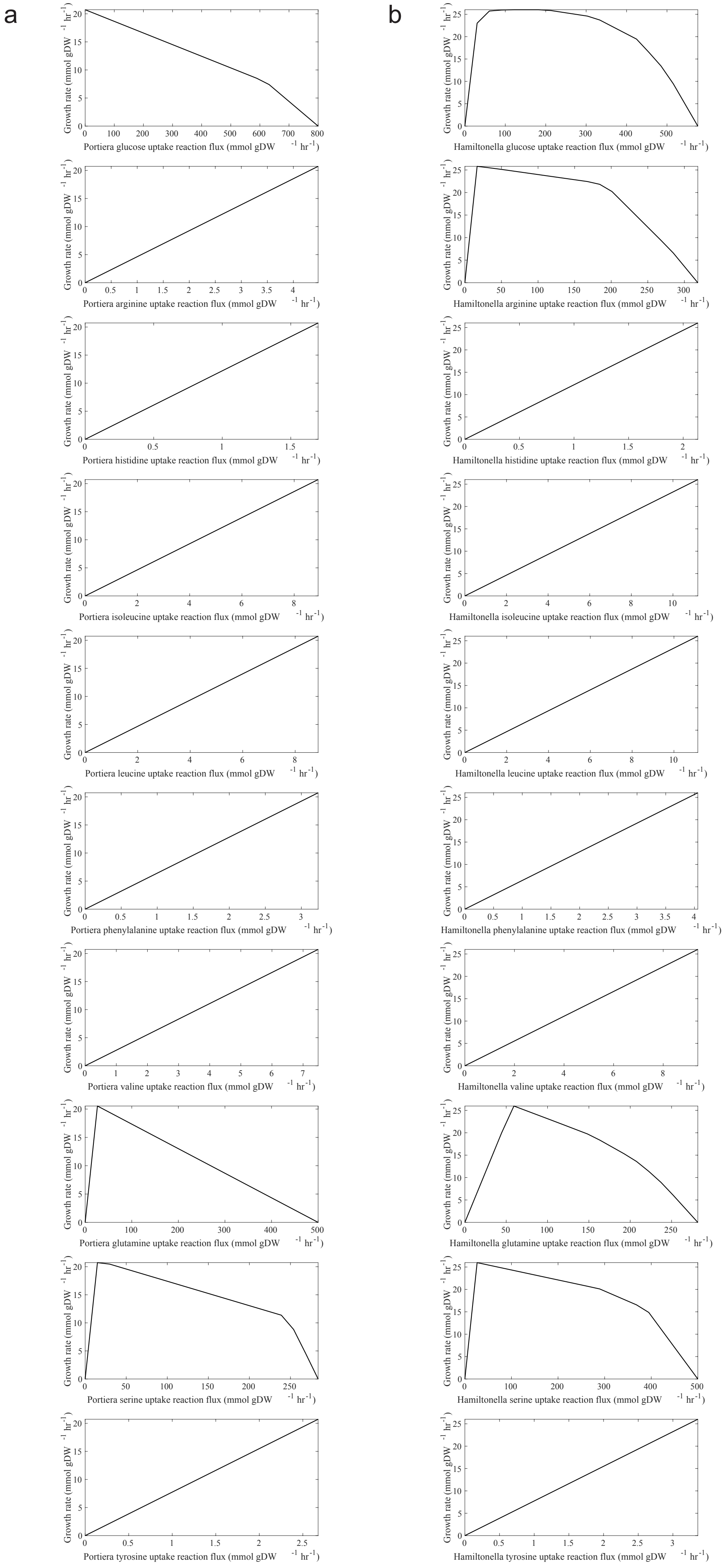

Figure S3. Effect of varying concentrations of shared metabolites on a) *Portiera* and b) *Hamiltonella* biomass production.

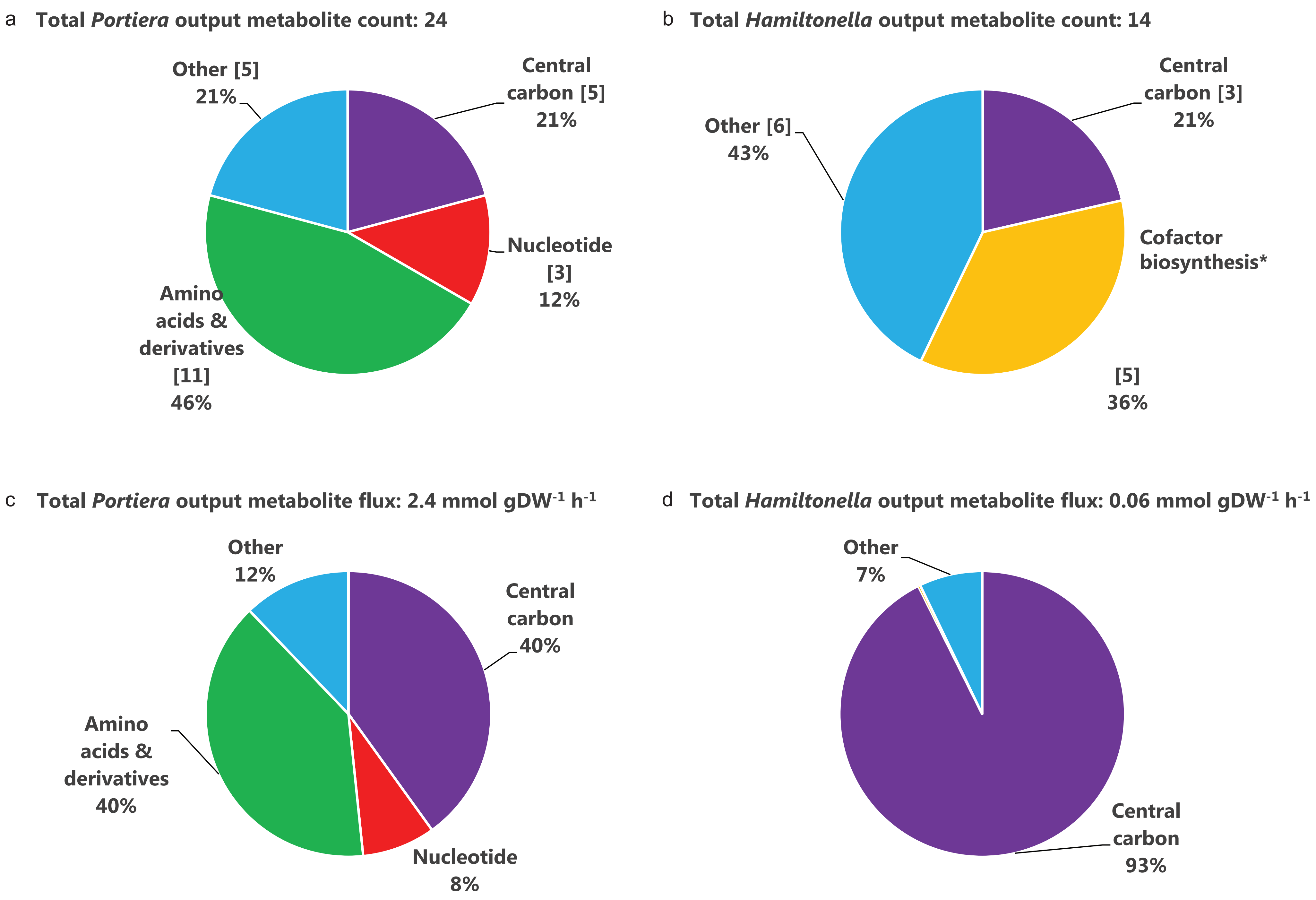

Figure S4. Metabolic outputs from bacterial compartments of 3-species integrated metabolic model Percentage composition of output metabolite classes based on metabolite counts for a) *Portiera* and b) *Hamiltonella* and metabolite fluxes for c) *Portiera* and d) *Hamiltonella*. Metabolite counts for each compound class are shown in parenthesis. Total metabolite count and fluxes out of each bacterium are indicated on top of each pie chart. Details of individual metabolite count and fluxes are provided in Table S7b. *Metabolite class includes cofactors, intermediates and side products of cofactor biosynthesis.

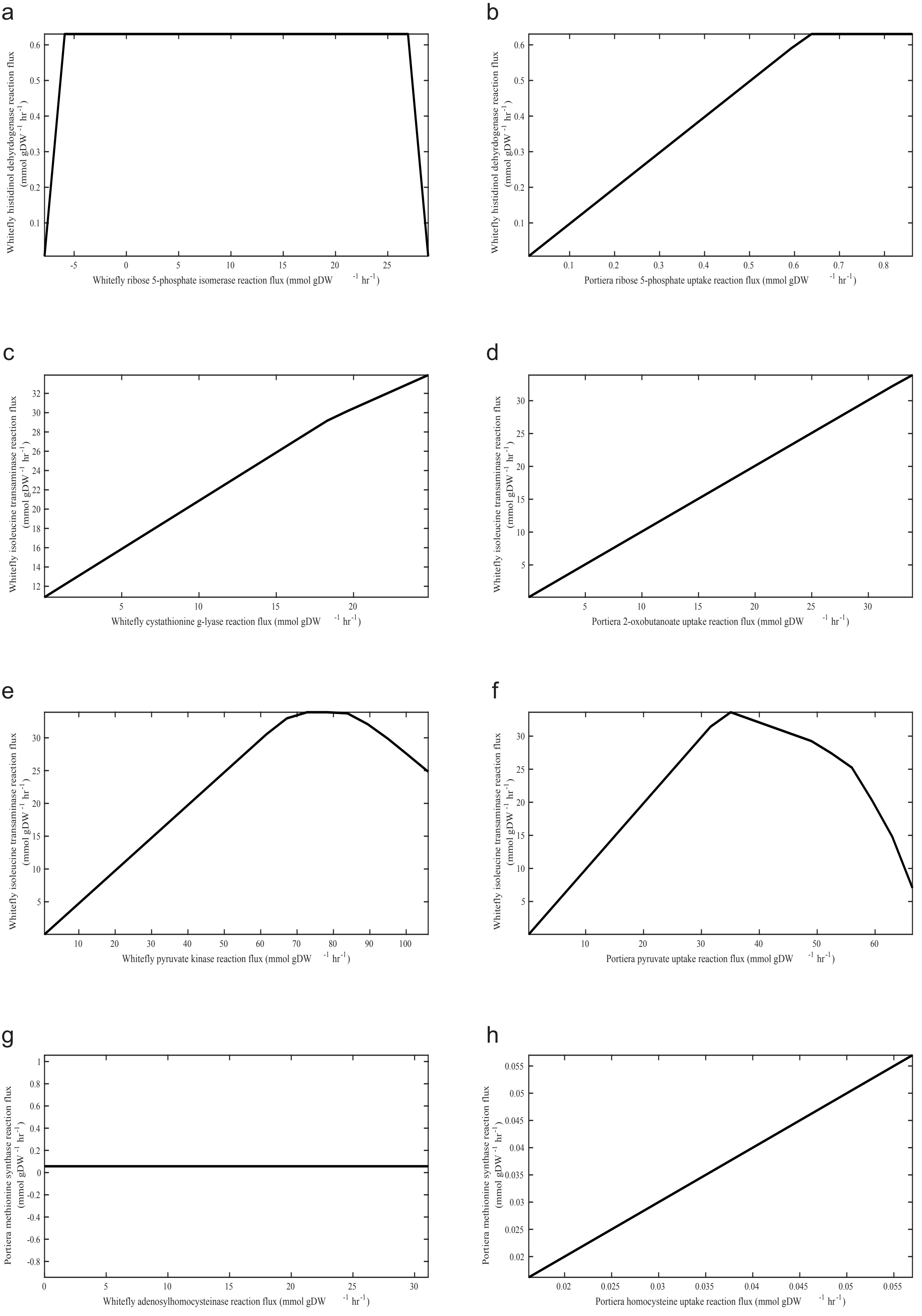

Figure S5. Effect of host precursor synthesis and precursor uptake rate by Portiera on the synthesis of EAAs.

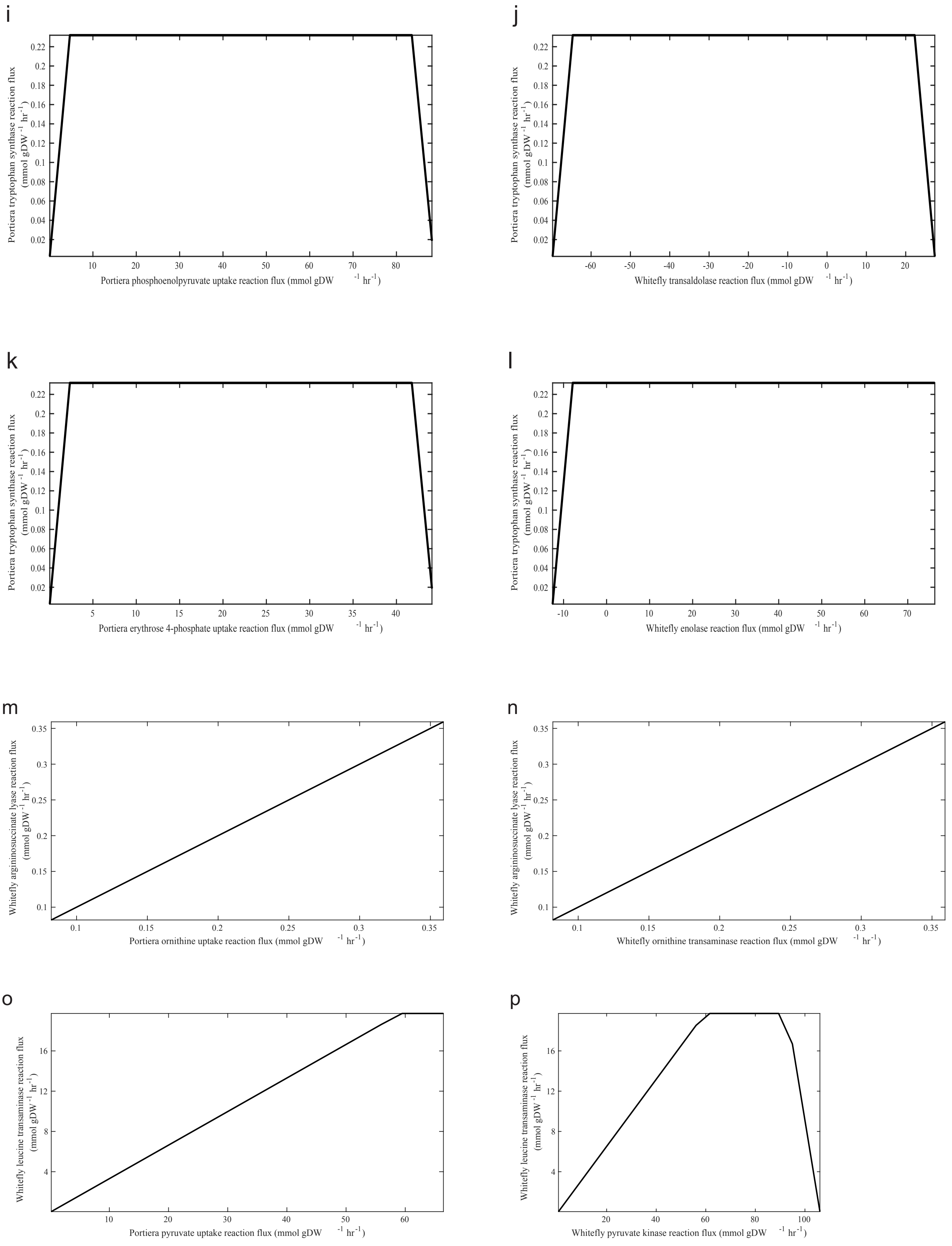

Figure S5. Effect of host precursor synthesis and precursor uptake rate by *Portiera* on the synthesis of EAAs..

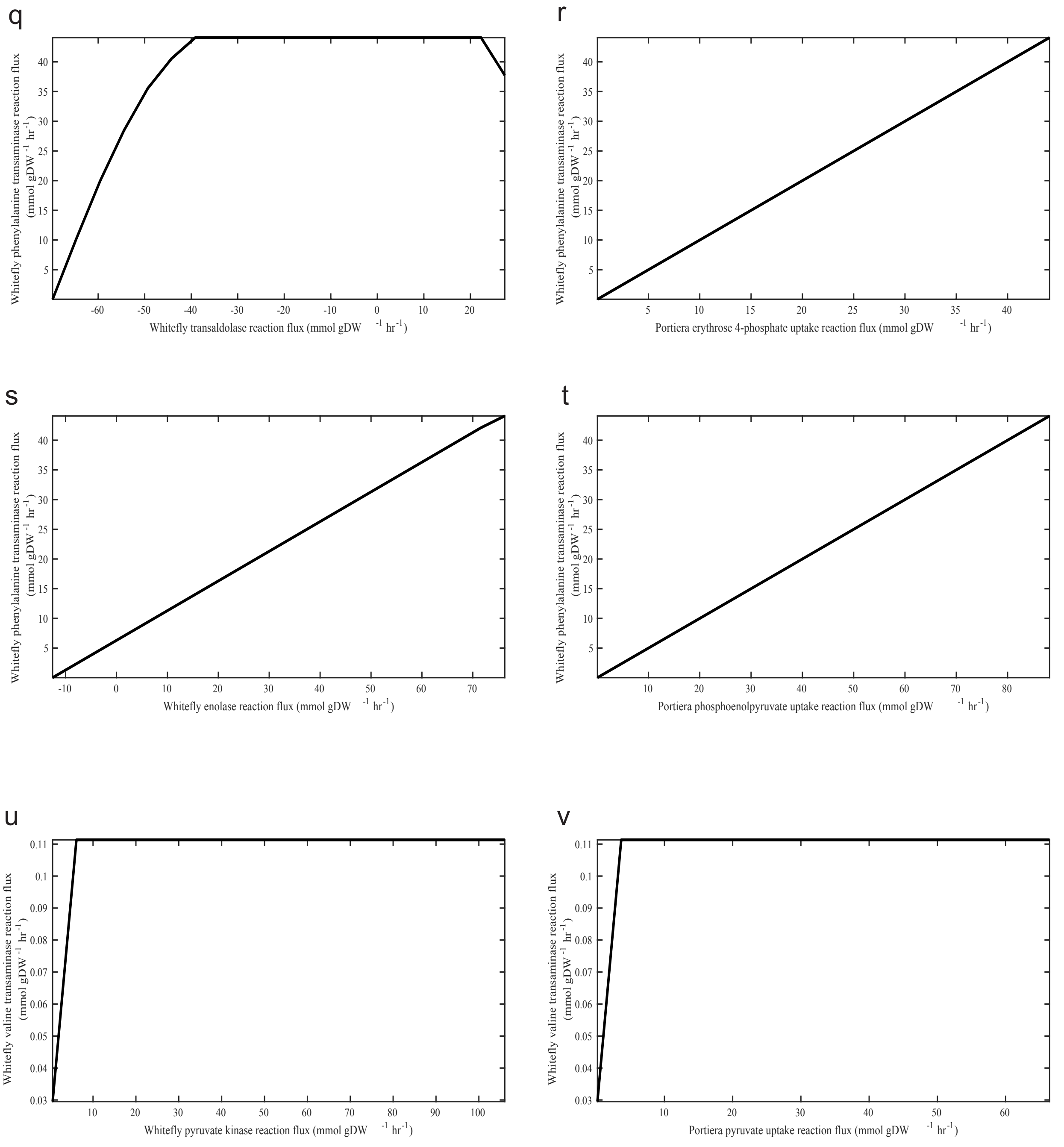

Figure S5. Effect of host precursor synthesis and precursor uptake rate by *Portiera* on the synthesis of EAAs.
